# Visualizing pathogens: Disfluent shapes of pathogens increase their perceived complexity and danger while realism and disfluency boost the credibility of visualizations

**DOI:** 10.1002/hbe2.232

**Published:** 2020-11-25

**Authors:** Alexander Skulmowski, Günter Daniel Rey

**Affiliations:** ^1^ Psychology of Learning with Digital Media Chemnitz University of Technology Chemnitz Germany

**Keywords:** computer‐generated imagery, COVID‐19, credibility, fluency, halo effect, health communication, judgment, realism, science communication, visualization

## Abstract

During the COVID‐19 pandemic, the public was regularly presented with visualizations of the viral pathogen causing this disease. Since there are several ways of visually communicating information, we investigate whether different types of visualizations affect how viewers judge the credibility of information as well as the complexity and potential harm of pathogens. A first experiment was conducted to assess whether a round, fluent shape elicits a different response than pathogens featuring disfluent components such as thread‐like appendages. Visualizations of disfluent bacteria were rated as more credible than those of fluent bacteria. In Experiment 2, bacteria were either presented as realistic renderings or as cartoon‐like line drawings (varied between‐subjects). Furthermore, half of the six bacteria had fluent shapes, while the other half featured disfluent shapes, resulting in the within‐subjects factor of fluency. Participants were asked to rate the credibility, complexity, and risk of serious illness associated with these bacteria. We found that disfluent bacteria were perceived as having a more complex metabolism and as holding a higher risk for serious illness. Furthermore, realism and disfluent shapes increase the credibility of visualizations, but not the credibility of additional information. These results have important implications for the field of science communication.

## 1 INTRODUCTION

Accurately communicating the risks of pathogens for human health has become an important issue during the COVID‐19 pandemic. During the outbreak of novel diseases, societies need to make tough decisions based on a limited knowledge base. Therefore, the means used to communicate this knowledge must be appropriate. The visual design of information plays an integral part in this endeavor. When supplying the public with information regarding a new pathogen, there are several possibilities for the design of visualizations that range from microscopic photographs and realistic renderings to simplified line drawings. Furthermore, these visualizations can feature different kinds of shapes, such as round shapes that are commonly preferred over spiky shapes due to appearing less harmful (Bar & Neta, 2006). In two experimental studies, we investigate whether the realism and the shapes of pathogens affect viewers' judgments of credibility, complexity, and the risk of serious illness.

### 1.1 Perceptual fluency and complexity as the basis of judgments

To understand the strong effects that visual design can have on judgments, we first need to consider theories of aesthetics and perception. An important theoretical approach toward perceptual and aesthetic judgments is fluency theory (for an overview, see Palmer, Schloss, & Sammartino, 2013). Fluency is described as a state in which viewers can easily process visual stimuli due to their ease‐of‐perception (e.g., Reber, Winkielman, & Schwarz, 1998; for an overview on processing fluency, see Reber, Schwarz, & Winkielman, 2004). For instance, it was shown that fluent, round shapes are aesthetically preferred over disfluent, angular shapes (e.g., Silvia & Barona, 2009). Disfluent shapes have been shown to trigger a sense of threat, perhaps due to associations of spiky and irregular shapes with needles or other objects that can cause pain or discomfort (Bar & Neta, 2006). In addition to these effects, disfluency has been associated with a higher memorability of stimuli (Diemand‐Yauman, Oppenheimer, & Vaughan, 2011). In a series of studies, a less easily readable (disfluent) font promoted higher recall scores of word stimuli than fluent fonts (Diemand‐Yauman et al., 2011). Similar effects have been found for disfluency induced by degrading the image quality of visualizations (Eitel, Kühl, Scheiter, & Gerjets, 2014). In the context of learning and memory research, these kinds of effects are called *desirable difficulties*, as they act as design factors that make people more carefully consider information (Bjork, 1994).

Even beyond basic perceptual aspects, there is evidence that disfluency can alter even more complex (social) judgment processes such as stock market predictions (Alter & Oppenheimer, 2006) and xenophobia (Rubin, Paolini, & Crisp, 2010; for an overview, see Alter, 2013). For instance, disfluency can affect people's confirmation bias (Hernandez & Preston, 2013). In a series of studies, disfluency induced cognitive difficulties that fostered a more deliberate cognitive mode, helping to persuade participants of a different viewpoint (Hernandez & Preston, 2013). Based on these results, the importance and potentials of (dis‐)fluency for science communication become clear.

A factor involved in visual fluency lies in the characteristic of complexity (Ball, Threadgold, Marsh, & Christensen, 2018), often determined by the number of different elements and their arrangement (e.g., Orth & Wirtz, 2014). The complexity of an image is thought to be a cause of the impression of visual disfluency (Ball et al., 2018; Sohn, Seegebarth, & Moritz, 2017). Complexity can prevent fluent visual processing and thus can induce negative affect (Orth & Wirtz, 2014). In an eye‐tracking study, Orth and Crouch (2014) demonstrated that complex packaging designs negatively affect the attractiveness in a retail context by being distracting. Similarly, in the case of interior design for stores, it was found that complexity also decreases ratings of attractiveness (Orth & Wirtz, 2014). Judging from these results, it is evident that complexity can profoundly affect judgments, often in a negative way.

Our primary interest lies in judgments concerning properties that are not directly related to the immediate (visual) impression. Such far‐reaching biases of impressions on judgments are known as *halo effects* and generally consist in changes in judgments based on unrelated properties (Nisbett & Wilson, 1977). As an example of such an effect, one study found that men rate more attractive female authors as more talented (Kaplan, 1978). In the case of pathogens, we assumed that disfluency will have a halo effect concerning the complexity of the metabolism of these pathogens. Disfluent pathogens may elicit a higher perceived metabolism complexity that may be associated with a negative response, similar to the studies summarized above. In the case of pathogens, we chose the risk for serious illness as a negative outcome that may be increased by the visual cue of disfluency and the associated perceived complexity of the stimulus. In sum, we assume that disfluency gives viewers the impression of a more complex metabolism. This perceived complexity may induce a halo effect that increases the perceived threat of pathogens.

### 1.2 Realism as a type of disfluency and the effect on credibility

In addition to the visual properties of fluency and complexity, a third characteristic of visualizations is known to affect the impression of credibility. Zanola, Fabrikant, and Çöltekin (2009) have shown that the credibility of visualizations can depend on their degree of realism. They compared three different visualizations of a city that either featured the style of a hand‐drawn line sketch, a rather geometric technical CAD drawing, and a highly detailed realistic rendering (Zanola et al., 2009). They found significant differences between the credibility ratings with a directional pattern indicating that a higher realism leads to higher ratings of credibility (Zanola et al., 2009). Based on these results, we assume that the rendering style of pathogen visualizations will affect credibility ratings.

Realistic visualizations and their high number of details have been presented as a form of visual disfluency (Skulmowski & Rey, 2018, 2020b). Realistic details can be thought of as disfluent visual components as they can interfere in the processing of shapes (Skulmowski & Rey, 2018). Due to this kinship between the two style characteristics, we assumed that realism may further enforce the effects of disfluency by amplifying the disfluent properties of the shape of a pathogen. The aspect of realism was investigated in Experiment 2.

## 2 EXPERIMENT 1

For both studies, we chose to use bacteria as the experimental stimuli due to their variety of shapes. Besides, the ongoing COVID‐19 pandemic might have biased participants' judgments due to a potentially higher generalized fear of viruses and therefore led us to choose bacteria as a more neutral set of stimuli. Given the strong negative associations with disfluent stimuli outlined above, we assumed that this will affect judgments concerning the helpfulness and harm of bacteria. It is known that people often attribute negative intentions to agents they perceive as harmful, even if their actions are beneficial. People tend to judge negative side effects as intentional, whether positive side effects as nonintentional (Knobe, 2003; see Feltz, 2007, for an overview). In line with this reasoning, we figured that there may be a similar asymmetry in judgments concerning the helpfulness and harmfulness of bacteria that might be exacerbated by disfluency. We hypothesized that disfluent bacteria would lead to particularly high ratings of harmfulness and lower ratings of helpfulness (Hypotheses (H1a) and (H1b)). In addition to this interaction effect, we were interested in whether the fluency of visualized bacteria affects the credibility of the visualization. In line with research that showed an increased credibility for more detailed visualizations of a city (Zanola et al., 2009), we assumed that a visualization featuring disfluent details would lead to higher ratings of credibility than a fluent visualization (Hypotheses (H2a) and (H2b)).

### 2.1 METHODS

#### 2.1.1 Participants and design

The study used a repeated‐measures design. All participants viewed two different bacteria, one of them fluent and the other one disfluent in design, and gave credibility ratings. For the analysis of harmfulness, all participants gave two ratings (usefulness and harmfulness) that were used as a second within‐subjects factor besides fluency, leading to a 2 × 2 design.

A previous study investigating the confidence in visualizations differing in their level of realism resulted in a very large effect of *η*_p_^2^ = .71 (Zanola et al., 2009). However, we chose to align our power calculation for the smallest effect of interest with sample sizes used in recent research on the cognitive processing of realistic visualizations (e.g., Skulmowski & Rey, 2020a). We planned to continue data collection until at least 40 participants had completed the study based on a power calculation for within‐subject factors using G*Power (Version 3.9.1.2; Faul, Erdfelder, Buchner, & Lang, 2009) with *α* = .05, power = .80, correlation between measures = .5, and *η*_p_^2^ = .05. Forty‐four participants completed the study before we stopped the data collection. From these 44, three participants were removed from all analyses due to their responses to data quality control questions (see below), leaving *n* = 41 as the sample for the analyses. In line with participation requirements, the participants were aged between 18 and 30 years, were German native speakers, had no to little knowledge concerning bacteria and their effects on humans, and took part using a computer or laptop. Also, 40 participants were students of media‐related university courses, 27 were female. Participants were offered to gain partial course credit for their participation. The study was advertised using a university mailing list.

#### 2.1.2 Materials and procedure

**FIGURE 1:**
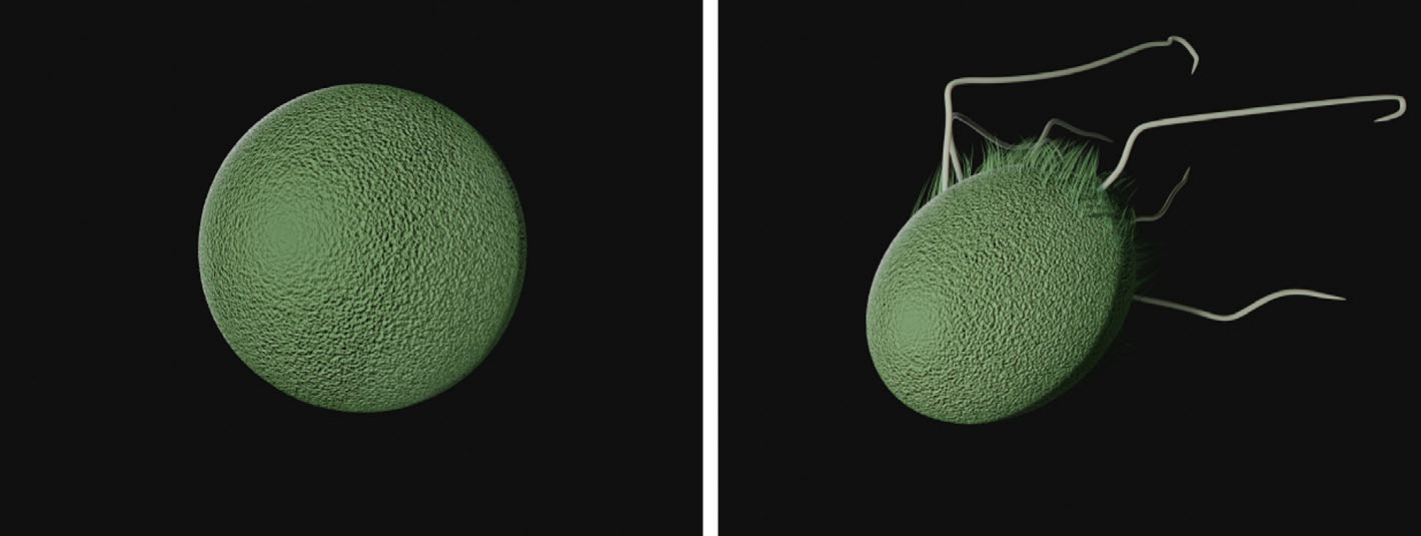
The stimulus materials used for Experiment 1. The left panel shows a fluent bacterium, the right panel depicts a disfluent bacterium

After providing informed consent, participants were asked to indicate their gender and their course of study. On an instruction page, participants were informed that they would learn information regarding two kinds of bacteria and that they should carefully read the information. A brief text reminded participants that bacteria can have both positive and negative functions in the human body. On the next two pages, participants were shown two different computer‐generated images of bacteria (see Figure 1). The order of the two pages was randomized. The shape of the fluent bacterium was perfectly round and, thus, fluent by definition. The disfluent bacterium featured several thread‐like appendages and short hair‐like strands that do not follow fluent lines. The images were inspired by illustrations from the Public Health Image Library (PHIL; https://phil.cdc.gov/) of the Centers for Disease Control and Prevention (CDC). We used Blender 2.82a (https://www.blender.org) to create the images.

Alongside the images, participants were presented with one helpful and one harmful effect of each bacteria (for instance, “Can remove and weaken toxins” and “Can cause blindness”). These statements (two positive and two negative effects) are based on Parker, Schneegurt, Tu, Lister, and Forster (2016) and were included to make participants aware of the fact that bacteria can have both positive and negative effects. The information was randomly compiled for each participant from two lists with two effects. Participants were informed that the presented materials may contain errors at the beginning of the experiment and were reminded of this fact in the instructions and the debriefing text presented at the end of the study.

Below each bacteria, participants were asked to respond to whether they were convinced that the visualization portrays the bacterium in an accurate way on a 7‐point scale (ranging from *I do not agree at all* to *I fully agree*). This approach was based on Zanola et al. (2009). After viewing both pages, participants completed a 1‐min filler task (sorting task) and were presented with two additional pages that featured the two bacteria again in a newly randomized order. On these pages, participants were asked to indicate (a) how useful and (b) how harmful the given bacteria are for the human body on 7‐point scales (ranging from *not useful/harmful at all* to *very useful/harmful*).

On the following page, participants were asked to indicate whether they encountered major technical difficulties and whether they were strongly distracted during the study as quality control items by responding “yes” or “no” (following Skulmowski & Rey, 2020b). The study was conducted online using SosciSurvey (Version 3.2.05‐i; https://www.soscisurvey.de).

### 2.2 RESULTS

**FIGURE 2:**
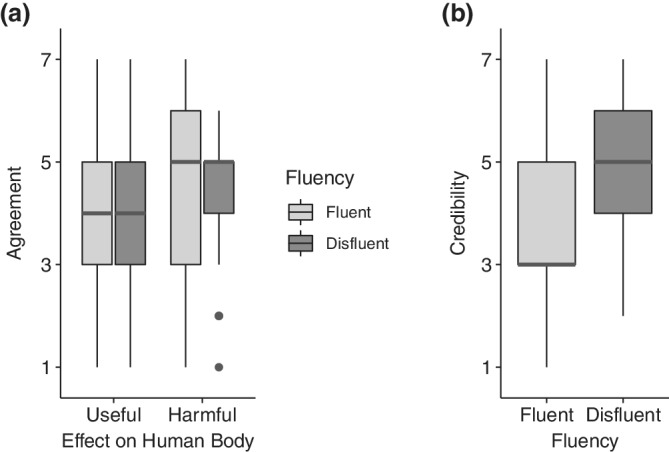
Descriptive results of Experiment 1. All dependent variables were rated on 7‐point scales with a minimum of 1 and a maximum of 7). (a) Boxplot of the usefulness and harmfulness of the bacteria based on their fluency. (b) Boxplot of the credibility of the visualizations based on fluency

Both dependent variables were analyzed using analyses of variance (ANOVAs). As the assumption of normality of residuals was violated for both ANOVAs, we used a nonparametric aligned rank transformed (ART) method (Wobbrock, Findlater, Gergle, & Higgins, 2011). The untransformed data are presented in Figure 2. A significance level of *α* = .05 is assumed for all analyses in this paper.

#### 2.2.1 Helpfulness and harm

A 2 × 2 repeated‐measures ART ANOVA with the factors fluency (fluent vs. disfluent) and response type (helpfulness rating vs. harmfulness rating) revealed no significant interaction effect (*p* = .775; see Figure 2a). Hypotheses (H1a) and (H1b) were not confirmed by the data.

#### 2.2.2 Credibility

Using a repeated‐measures ART ANOVA with the factor fluency (fluent vs. disfluent), we found a significant main effect, *F*(1, 40) = 11.41, *p* = .002, *η*_p_^2^ = .22. As can be seen from the data (see Figure 2b), the disfluent visualization elicited higher ratings of credibility (see Figure 2b). Therefore, Hypotheses (H2a) and (H2b) were confirmed.

## 3 EXPERIMENT 2

In a second study, we sought to assess the cognitive mechanism behind judgments of credibility and risk. As presented in the introduction, our proposed model is based on the assumption that a more disfluent shape triggers the impression of a more complex metabolism. This higher complexity may lead to a halo effect that increases the perceived threat of bacteria. Also, we assess whether the credibility ratings are biased by another halo effect of disfluency and realism (based on Zanola et al., 2009). In particular, we use additional question items to measure whether this halo effect is strong enough to influence the credibility of supplemental factual information. As outlined in the introduction, it is plausible to assume that realism is related to disfluency due to the potential of realistic details for breaking up the visual flow (see Skulmowski & Rey, 2018). Hence, we assumed that realism will strengthen the effects of disfluency on ratings of complexity and risk.

Hypothesis (H1a) A higher level of realism leads to higher ratings of credibility than fluent shapes.

Hypothesis (H1b) *Disfluent shapes lead to higher ratings of credibility than fluent shapes*.

Hypothesis (H2a) *Disfluent shapes lead to higher ratings of metabolism complexity than fluent shapes*.

Hypothesis (H2b) *Realism amplifies the effect of disfluent shapes on complexity ratings*.

Hypothesis (H3a) *Disfluent shapes lead to higher ratings of risk of serious illness than fluent shapes*.

Hypothesis (H3b) *Realism amplifies the effect of disfluent shapes on ratings of risk of serious illness*.

### 3.1 Methods

#### 3.1.1 Participants and design

The experiment uses a split‐plot design with the between‐subjects factor of realism (schematic vs. realistic; block‐randomized) and the within‐factor of fluency (fluent vs. disfluent). The within‐subjects factor has six items, requiring a linear mixed‐effect model for the analysis. Again, we used sample sizes of current research as a guide for the smallest effect of interest (Skulmowski & Rey, 2020b). The target sample size of 50 was calculated using G*Power 3.1.9.2 by assuming an interaction effect in a 2 × 2 mixed repeated‐measures ANOVA (*α* = .05, power = .80, correlation between measures = .5, and *η*_p_^2^ = .04). The data of 53 participants were collected and the data of three participants were removed for reporting distractions or technical difficulties.

These 50 participants fulfilled the same criteria as listed in Experiment 1. Thirty‐six were female and 46 were students in a media‐related university course. Partial course credit was offered as an incentive. The experiment was advertised using a university mailing list.

#### 3.1.2 Materials

**FIGURE 3:**
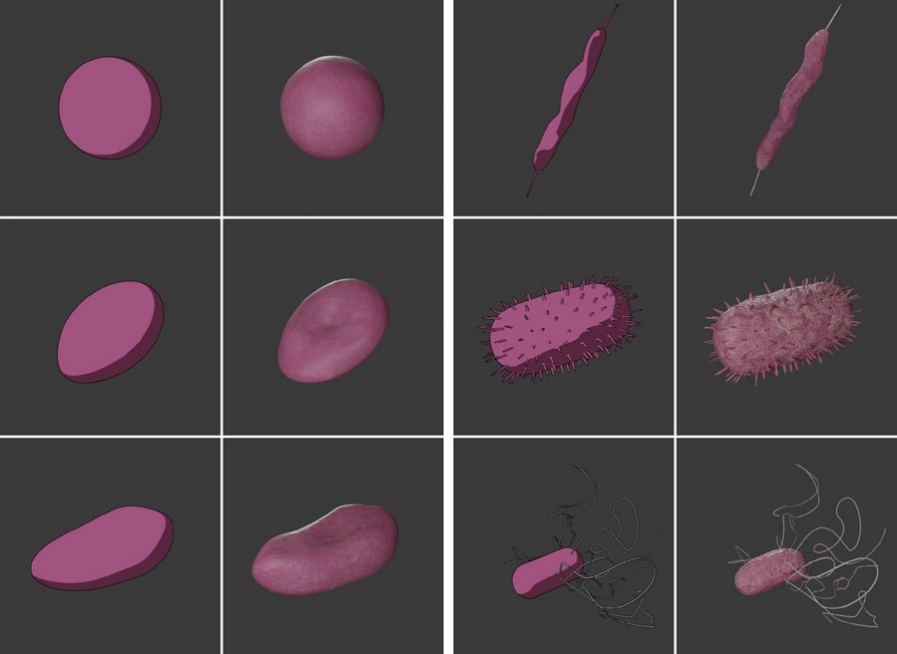
Stimulus sets for Experiment 2. The two columns on the left show the fluent bacteria while the two columns on the right feature visualizations of disfluent bacteria. Each visualization was produced in a toon‐like style with an outline and minimal shading (shown on the left side of the column pairs) and a realistically rendered version (presented on the right side of each column pair)

To have high statistical power, we used six visualizations of bacteria for this study (see Figure 3). Again, we based our designs on references taken from the PHIL database provided by the CDC and used Blender 2.82a to generate the visualizations. The three fluent bacteria have flowing, round shapes while the disfluent bacteria all feature one or more spiky or otherwise disfluent elements. Besides, the rendering style of the bacteria was varied. Participants either saw the bacteria as a cartoon‐like rendering with simple shading and an outline or as a realistic rendering with more details, textures, bump maps, and more elaborate lighting.

Each of the six bacteria was presented on a page together with two symptoms, one mild (such as cough or sore throat) that usually does not require treatment and one severe symptom (such as paralysis or kidney failure) that usually requires medical treatment. These symptoms were presented to give participants a shared frame of reference to base their later judgments on. The symptoms were randomly drawn from two lists of six symptoms for each participant. The information was based on information found in Parker et al. (2016). As in Experiment 1, participants were made aware of the potential for incorrect information at various stages of the study.

Below the bacteria visualizations, participants were presented with several survey items. They were asked to respond to three question items concerning the credibility of the presented information (see Table 1). Participants were asked to respond to these items on 7‐point scales (ranging from *I do not agree at all* to *I fully agree*). These six surveys all had a *ω* of at least .84. Furthermore, participants were asked to respond to a question on how complex they think the metabolism of the respective bacterium is on a 7‐point scale (ranging from *very simple* to *very complex*). At a later stage of the study, participants were asked to estimate the probability of a serious illness caused by an infection with the bacteria on a slider ranging from *very low* to *very high*.

**TABLE 1:** Credibility survey items used in Experiment 2 (English translation in the left column, German original in the right column)

I perceive the information as credible.	Ich empfinde die informationen als glaubwürdig.
I am convinced that the facts concerning the bacterium are correct.	Ich bin überzeugt, dass die Fakten zum bakterium korrekt sind.
I am convinced that the visualization portrays the bacterium in an accurate way.	Ich bin überzeugt, dass die abbildung das bakterium akkurat darstellt.

#### 3.1.3 Procedure

The overall procedure was similar to Experiment 1. After the consent page and questions concerning the gender and course of study, there was a short instruction. In the learning phase, participants viewed the six bacteria on individual pages that included the three credibility items and the question concerning the complexity of the respective bacteria's metabolism. These order of these pages was randomized per participant. After a filler task, the six bacteria were presented again on six pages that were newly randomized per participant. On these pages, participants were asked to indicate the risk of serious illness. Finally, participants were asked regarding technical difficulties and distractions. The experiment was conducted as an online study using SosciSurvey (Version 3.2.05‐i; https://www.soscisurvey.de).

### 3.2 RESULTS

**FIGURE 4:**
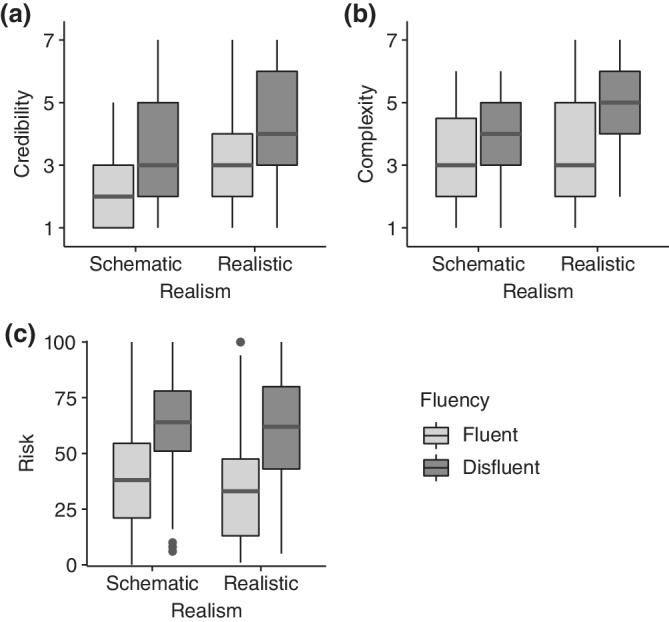
Descriptive results of Experiment 2. (a) Boxplot of the untransformed credibility ratings of the visualization item of the survey (7‐point rating from 1 to 7). (b) Boxplot of the untransformed complexity ratings (7‐point rating from 1 to 7). (c) Boxplot of the untransformed risk ratings (0–100 rating of probability)

Analyses were performed using linear mixed‐effect models (Bates, Mächler, Bolker, & Walker, 2015). We used the analysis approach and formulas for split‐plot designs presented by Baayen, Davidson, and Bates (2008) including model selections based on likelihood‐ratio tests. For the reported models, assumption tests using the Shapiro–Wilk test and Levene's test performed on the model residuals did not reach significance, indicating suitability of the analyses. The untransformed data are presented in Figure 4.

#### 3.2.1 Credibility

The ratings obtained using the three‐item survey were analyzed using the fixed effects of realism (schematic vs. realistic) and fluency (fluent vs. disfluent). The mixed‐effect model included random intercepts for subjects and stimuli and was selected using a likelihood‐ratio test. No effect reached significance, although the main effect of fluency nearly became significant (*p* = .059). Thus, we know that the realism and fluency of the visualizations did not significantly alter the overall credibility ratings of the information, indicating a lack of a halo effect. We proceeded by analyzing the data of the image‐related question item already used in Experiment 1 separately. In this analysis, we used the same two fixed effects, but by‐subject and by‐stimulus random intercepts as well as by‐subject random slopes for fluency. Both realism and fluency significantly increased the credibility ratings for the images, *t*(48) = −2.31, *p* = .025, and *t*(4.85) = 3.39, *p* = .020, respectively (see Figure 4a). Thus, we conclude that Hypotheses (H1a) and (H1b) were partially confirmed. Although the visual characteristics of the images did not increase the credibility of the verbal information presented alongside the visualizations, the credibility of the images benefited from a more realistic and disfluent style.

#### 3.2.2 Complexity

The mixed‐effects model for the complexity ratings used the fixed effects of realism and fluency. By‐subject and by‐stimulus random intercepts as well as by‐subject random slopes for fluency were utilized. In accordance with Hypothesis (H2a), disfluent shapes elicited significantly higher ratings of complexity, *t*(5.54) = −3.74, *p* = .011 (see Figure 4b). Hence, the halo effect of shape disfluency on ratings of metabolism complexity was confirmed empirically. However, realism did not increase this effect as indicated by a nonsignificant interaction effect (*p* = .227), contrary to Hypothesis (H2b).

#### 3.2.3 Risk for serious illness

The mixed‐effect model for the ratings of risk for serious illness had the same structure as for the complexity ratings. As predicted in Hypothesis (H3a), disfluency increased the risk ratings for serious illness significantly, *t*(9.86) = −5.82, *p* < .001 (see Figure 4c). There was no interaction effect between realism and disfluency (H3b), *p* = .766.

## 4 GENERAL DISCUSSION

In two experiments, we assessed the influence of disfluency and realism on judgments concerning the credibility, complexity, and health threat of bacteria. Experiment 1 revealed that disfluent visualizations of bacteria are rated as more credible than fluent visualizations. The results of Experiment 2 might explain this effect. Illustrations of disfluent bacteria give viewers the impression of a more complex metabolism. Since disfluent shapes are associated with threat (Bar & Neta, 2006), viewers most likely understand this complex metabolism as a greater threat for their health, leading to higher risk ratings of serious illness for these disfluent bacteria. While we found an increase in credibility ratings for disfluent and more realistic renderings compared with fluent and schematic visualizations (in line with the results regarding realism reported by Zanola et al. (2009)), these effects were not strong enough to cause a halo effect that increases the credibility of information presented alongside the illustrations.

The two studies offer further evidence for the importance of fluency in visual design. As previous studies found that sharp, disfluent shapes can induce a sense of threat (Bar & Neta, 2006), we show that this design aspect can be transferred to science communication. The more threatening‐looking shapes increased risk ratings in our study, but did not affect ratings of harmfulness. This contradicting result pattern may be explained by participants not attributing an intention to harm to the bacteria, therefore not resulting in differences in ratings of harm. Besides, disfluent shapes were rated as more credible. Perhaps, viewers assume that a more detailed visualization has been produced more carefully and thus may have a higher chance of being a correct representation. These results offer important cues for further research on disfluency.

One important implication is that science communicators should primarily consider the disfluency of visualizations to accurately convey the health risks associated with a pathogen. To accurately inform the public regarding the risks of pathogens, the level of disfluency should be appropriately chosen for the actual health risk to avoid panic reactions. Conversely, the awareness of serious health risks may depend on enough disfluent elements in pathogen visualizations. Also, we recommend using more realistic and more disfluent styles to enhance the credibility of visualizations.

Interestingly, we did not find evidence for hypotheses concerning additive effects of realism and disfluency. Realism did not substantially increase the credibility or perceived health risks of disfluent bacteria. Hence, it is plausible that people only use a very superficial heuristic focused on the overall shape when making such judgments and do not include more nuanced aspects such as the level of realism. This aspect should be considered in future research.

Our results have important implications for the use of visualizations in the field of health communication, in particular during the on‐going COVID‐19 pandemic and other health emergencies. To adequately inform the public regarding threats posed by pathogens, the visual design should be a major concern. As scientists, journalists, and governments have several options when choosing visualizations for press releases, news reports, and papers, we emphasize that the visual style of these (often computer‐generated) visualizations can strongly affect the assessment of a pathogen's health threat. Therefore, stronger consideration of this factor needs to be made to appropriately and responsibly communicate this risk to the public. As discussed by Slater et al. (2020), the ethical use of realistic graphics (particularly in the context of virtual reality and similar technologies) is an important emerging topic. As realism can be used as a means to persuade people (Slater et al., 2020), the design aspects investigated in our studies should be utilized with care. A broader debate of this ethical issue in the context of the COVID‐19 pandemic and the ensuing changes in the interplay between humans and technology (see Yan, 2020) will be necessary.

### 4.1 Limitations and outlook

It needs to be acknowledged that in a more typical situation in which information regarding pathogens is presented, more facts and information is likely to be presented alongside visualizations. However, our studies accurately capture settings in which only a limited amount of information is provided, for example, in a short online video clip or a brief magazine article. Still, future studies should examine if our effects stay robust if a larger number of facts is provided alongside visualizations. While we chose a more artificial experimental procedure that involved randomly combined information, it has to be seen whether these effects can be replicated using other materials. Future studies using more authentic materials should be conducted and could determine the mechanism behind our effects in greater detail.

It also needs to be considered that repeated exposure may change viewers' responses to images (Cox & Cox, 2002). Potentially, the effects of disfluent shapes on viewers vary depending on the viewing frequency of pathogen visualizations. Other design factors that have been shown to affect judgments could be used to shape viewers' responses to pathogen visualizations.

Further consideration should be given to the potential of realistic visualizations to be more memorable than more abstract representations (Skulmowski & Rey, 2020a). In case of future pandemics or smaller outbreaks of pathogenic diseases, more detailed representations of the (usually rather amorphous shapes of) pathogens could help the public to distinguish between these different agents of disease.

Finally, it needs to be considered that the pathogens displayed in the studies were bacteria and not viruses. This aspect should be kept in mind when using these results as a base for science communication related to COVID‐19. However, as the studies were conducted during the pandemic, we assumed that the word “virus” would trigger a too great sense of threat and chose to use bacteria as materials.

### 4.2 CONCLUSION

Since governments and medical experts need to avoid both mass panics and obliviousness for (new) pathogens, it is of vital importance that appropriate visualizations of these pathogens are presented to the public. Our experiments show that disfluent shapes can be used to trigger the impression of complexity and elicit a higher perceived risk of serious illness. Furthermore, disfluency and realism increase the credibility of visualizations. These design aspects should be considered in the design, creation, and public dissemination of (computer‐generated) visualizations during emergencies such as the COVID‐19 pandemic.

## CONFLICT OF INTEREST

Both authors declare no conflicts of interest.

## Data Availability

Both authors elect to not share data.
